# Monthly administrations of milbemycin oxime plus afoxolaner chewable tablets to prevent *Angiostrongylus vasorum* infection in dogs

**DOI:** 10.1186/s13071-016-1773-1

**Published:** 2016-09-02

**Authors:** Wilfried Lebon, Eric Tielemans, Steffen Rehbein, Pascal Dumont, Stephen Yoon, Fredéric Beugnet, Philippe Jeannin, Diane Larsen, Lénaïg Halos

**Affiliations:** 1 Merial S.A.S, 29 avenue Tony Garnier, 69007 Lyon, France; 2 Merial GmbH, Kathrinenhof Research Center, 83101 Rohrdorf, Germany; 3 Merial, Inc, Duluth, GA 30096-4640 USA

**Keywords:** *Angiostrongylus vasorum*, Prevention, Dog, Afoxolaner, Milbemycin oxime

## Abstract

**Background:**

Infection of dogs with the cardiopulmonary nematode *Angiostrongylus vasorum* may result in severe clinical disease therefore adequate prevention is necessary. A randomized, negative control, blinded study was conducted to evaluate the efficacy in the prevention of canine *A. vasorum* infection after monthly administrations of NexGard Spectra®, a novel chewable tablet formulation combining the insecticide and acaricide afoxolaner and the anthelmintic milbemycin oxime, in a multiple challenge (trickle infection) model.

**Methods:**

Twenty beagle dogs were challenged orally with doses of approximately 32–43 third-stage larvae of *A. vasorum* once every other week on seven occasions (Study Days -7, 7, 21, 35, 49, 63 and 77). Ten dogs were administered NexGard Spectra® as close as possible to the minimum recommended dose of afoxolaner and milbemycin oxime, i.e. 2.5 mg/kg body weight and 0.5 mg/kg body weight, respectively, four times at monthly intervals (Study Days 0, 28, 56 and 84) while the remaining ten dogs served as untreated controls. For parasite recovery and count, dogs were euthanized humanely and necropsied six to eight days following the last treatment (Study Days 90–92). Beginning six weeks after first inoculation, faeces were collected on a bi-weekly basis and examined for first-stage larvae of *A. vasorum*.

**Results:**

Untreated dogs harboured 39–95 adult *A. vasorum* (geometric mean, 66.4), while zero to 24 adult *A. vasorum* were recovered from the treated dogs (geometric mean, 3.4; *P* < 0.0001). Thus, efficacy of NexGard Spectra® administered at monthly intervals against incoming *A. vasorum* was 94.9 %. Compared to the untreated controls, larval excretion of the treated dogs was reduced by 99.9 % (*P* < 0.0001).

**Conclusion:**

Results of this study demonstrate that NexGard Spectra®, when administered at monthly intervals, can effectively prevent canine *A. vasorum* infection.

## Background

Parasitic nematode infections of the cardiopulmonary organs of dogs have received increasing attention by both practising veterinarians and parasitologists in recent years. For more than two decades, canine angiostrongylosis has expanded its geographical distribution and appears to occur with increasing prevalence in Europe [1–3]. In certain regions of Denmark, Germany, Hungary, France, Ireland, Italy, the Netherlands, Poland, Portugal, Sweden and Switzerland, canine angiostrongylosis is considered to be endemic [1–5]. In the United Kingdom, hyper-endemic foci have been identified where practitioners reported more than 20 cases per year. Veterinary practices in those areas are 15 to 16 times more likely to see clinical angiostrongylosis than anywhere else [2, 3, 6].

Canine angiostrongylosis is caused by the nematode, *Angiostrongylus vasorum*. The parasite, first described in France in the 19th Century [7], is commonly known as “canine lungworm” or “French heartworm”. Adult *A. vasorum* reside in the right heart and pulmonary arterial tree of dogs and other canids. As most members of the superfamily Metastrongyloidea, *A. vasorum* has an indirect life-cycle with canids as the definitive host, terrestrial gastropods (mainly slugs) as intermediate hosts, and frogs acting as paratenic or intermediate hosts [8]. Adult female nematodes release eggs which are carried to the pulmonary capillaries where they are trapped. First-stage (L1) larvae hatching from the eggs enter the alveoli, pass through the tracheobronchial tree, pharynx, gastrointestinal tract, and are excreted in the faeces. Dog faeces are an attractive food for many gastropod molluscs [9] in which the larvae develop to become infectious for the final host. In common, with most metastrongyloid nematodes, *A. vasorum* seems to display little or no specificity with respect to their molluscan intermediate hosts [10]. Dogs become infected by ingestion of intermediate or paratenic hosts containing the infectious third-stage (L3) larvae. Following ingestion by a canid, larvae penetrate the intestinal wall, enter the visceral lymph nodes and are then carried via the hepatic portal vein to the heart and pulmonary arterial system where first immature worms arrive 10 days following infection and develop into mature adults [10, 11]. The prepatent period of canine *A. vasorum* infection appears to vary considerably [9]. However, in recently reported studies, excretion of L1 larvae was observed to commence in most dogs six to eight weeks after single challenge infection [12–14].

Angiostrongylosis may substantially impact the health of infected dogs and is potentially life-threatening. Infection of dogs with *A. vasorum* may be asymptomatic; however, this is not the rule [15]. The disease may result in variable clinical conditions, ranging from mild manifestations to severe forms. Cardio-respiratory signs including cough, exercise intolerance and loss of condition are the most common clinical manifestations, with coagulation disorders being less common but more likely to be fatal [3, 16]. The course of the infection is often chronic and subtle and therefore owner may become aware only when severe conditions such as coagulopathies or neurological disorders occur, with frequently fatal outcome [12].

In this context, effective measures to prevent the infection are of great interest for dog owners and veterinarians in areas where *A. vasorum* is endemic. Currently, only a few veterinary products are licensed for *A. vasorum* prophylaxis if used at monthly intervals.

The present study was conducted to evaluate the efficacy of monthly administrations of the macrocyclic lactone milbemycin oxime in the prevention of canine *A. vasorum* infection, when administered in a novel chewable tablet formulation in combination with the isoxazoline afoxolaner (NexGard Spectra®).

## Methods

This controlled study was conducted in compliance with the international Cooperation on Harmonisation of Technical Requirements for Registration of Veterinary Medicinal Products (VICH) Guideline (GL) 9, entitled Good Clinical Practice and was in general accordance to VICH GL7, “Efficacy of Anthelmintics: General Requirements” [17], VICH GL19, “Efficacy of Anthelmintics: Specific Recommendations for Canines” [18] and the “World Association for the Advancement of Veterinary Parasitology (WAAVP) guidelines for evaluating the efficacy of anthelmintics for dogs and cats” [19]. The protocol of the study has been reviewed and approved by the Merial Institutional Animal Care and Use Committee and was in compliance with the local animal welfare legislation. All personnel involved in collection of efficacy data, health observations and physical examinations were blinded to the treatment assignment of the animals.

### Animals and study design

This study included 20 purpose bred, healthy beagle dogs of both sexes. Dogs weighed 10.0–16.4 kg and were nine months old at study start. The animals were group-housed under identical conditions by sex and treatment group, except for the individual collection of faeces and post-treatment health observations. Dogs enrolled in the study were negative for *A. vasorum* L1 larvae prior to the first inoculation as confirmed by the examination of faeces using the Baermann-Wetzel larval migration technique [20] and were considered healthy and suitable for inclusion in the study by a veterinarian.

Prior to first treatment (Study Day -1), dogs were ranked based on decreasing body weight and randomly allocated to two groups of 10 dogs each, untreated (Control) and treated (NexGard Spectra®). Throughout the study, animals were observed at least once daily for general health.

The *A. vasorum* strain originated from a naturally infected dog in Denmark and was passaged in foxes and dogs. A batch of 100+/−2 *Helix aspersa* snails originated from a French breeding farm were injected each with approximately 1,500 first-stage *A. vasorum* larvae in a single inoculation. Snails were maintained in a specific crate within the research center facilities during the whole duration of the study. Infection of dogs started 6 weeks after snails injection to allow molting of first-stage *A. vasorum* larvae into infective third-stage *A. vasorum* larvae. Briefly, every other week on seven occasions, starting on study days -7, tissues of 3 or 4 snails were processed by peptic digestion to recover third-stage *A. vasorum* larvae. The larvae were separated from the digest suspension by sieving followed by sedimentation and cleaning with tap water. Dogs were inoculated orally using a syringe with approximately 32, 43, 40, 41, 35, 32 and 37 third-stage larvae of *A. vasorum* respectively on days -7, 7, 21, 35, 49, 63 and 77. The total inoculum size per dog was approximately 260 larvae.

### Treatment

The dogs were weighed on the day before each treatment for dose determination and any remaining food was removed on the day prior to treatment. Dogs allocated to the treated group were dosed orally on Study Days 0, 28, 56 and 84 with a combination of afoxolaner plus milbemycin oxime chewable tablets (NexGard Spectra®, Merial). Three sizes of chewable tablets were used (0.5 g, 1 g, and 2 g containing 9.375 mg + 1.875 mg, 18.75 mg + 3.75 mg, and 37.5 mg + 7.5 mg of afoxolaner plus milbemycin oxime, respectively) and combined as appropriate in order to achieve dosing of the dogs as close as possible to the minimum recommended dose of 2.5 mg/kg afoxolaner and 0.5 mg/kg milbemycin oxime.

All dogs were observed for adverse reactions hourly for four hours after each treatment administration.

### Faecal examination

Faeces were collected from each dog on Days 35, 49, 63, 77 and 90, and 10 g samples each were examined for the excretion of *A. vasorum* L1 larvae using the Baermann-Wetzel method.

### Clinical follow-up

Throughout the study, animals were observed at least once daily for general health. Physical examinations including determination of heart rate and respiration rate, auscultation of the lungs, inspection of mucous membranes (conjunctivae and inner lips) were conducted on Days 31, 55 and 87. In addition, body weight of all dogs was determined on Days -1, 27, 55, 83 and on the day of necropsy.

### Parasite recovery and count

On Days 90 to 92, dogs were euthanized humanely. *Angiostrongylus vasorum* were recovered from the heart and pulmonary arteries by reverse lung perfusion followed by dissection as described previously [12]. Liquids collected were strained through a 32 μm mesh size sieve in order to facilitate the collection of nematodes of various sizes. All *A. vasorum* were collected and counted (tails in the case of fragments). A macroscopical examination of the lungs was performed during necropsy.

### Statistical analysis

The primary endpoint for the treatment effectiveness claim was the total *A. vasorum* count including immature and adult worms. These counts were transformed to the natural logarithm of (count +1) for calculation of geometric means for each treatment group. Efficacy for the treated group was determined for *A. vasorum* by calculating the percent efficacy as 100[(C-T)/C], where C is the geometric mean among untreated controls and T is the geometric mean among the treated animals.

Individual faecal *A. vasorum* L1 larval counts of sampling Days 35, 49, 63, 77 and 90 were used to calculate the larval output over the course of the study (determination of period larval counts between two samplings using the trapezoidal rule summed up to estimate the cumulative larval output). Cumulative larval outputs were transformed as described above for calculation of geometric means for each treatment group.

For both *A. vasorum* worm counts and cumulative *A. vasorum* larval output, log-counts of the treated group were compared to the log-counts of the untreated Control Group using an F-test adjusted for the allocation blocks used to randomize the animals to the Treatment Groups. The MIXED procedure in SAS Version 9.4 was used for the analysis of variance, with the Treatment Groups listed as a fixed effect, and the allocation blocks listed as a random effect. All testing was two-sided at the significance level α = 0.05.

## Results

No adverse event or abnormal clinical reaction related to treatment was observed throughout the study, indicating that the monthly treatment with NexGard Spectra® was well accepted and safe. A mean dose of 0.58 ± 0.04 mg/kg of milbemycin oxime, the anthelmintic active in the chewable tablet formulation, was administered at each of the four treatment time-points.

### *Angiostrongylus vasorum* nematode counts

The counts of *A. vasorum* collected at necropsy are presented in Table 1. *Angiostrongylus vasorum* were recovered from all untreated dogs, with 39 to 95 adult worms recovered per dogs, corresponding to individual establishment rates ranging from 15 to 36.5 %. Dogs treated with NexGard Spectra® harbored 0 to 24 adult *A. vasorum*. The geometric mean worm count in the treated group (3.4) was significantly lower (*F*_(1,9)_ = 92.61, *P* < 0.0001) compared to the geometric mean worm count in the untreated control group (66.4), and efficacy of NexGard Spectra® administered at monthly intervals against incoming *A. vasorum* was 94.9 %.

**Table 1:** Nematode counts and percentage efficacy of monthly administered NexGard Spectra® against *Angiostrongylus vasorum*

Group	Dog ID	Nematode recovery at necropsy	
		*A. vasorum* count	Group geometric mean
Untreated control	57699	53	66.4
	57666	95	
	58147	93	
	58247	44	
	57870	62	
	58039	39	
	58043	75	
	58416	87	
	58192	91	
	58312	56	
NexGard Spectra®-treated	58286	24	3.4
	93641	3	
	94510	4	
	58036	6	
	91019	1	
	57856	2	
	58395	4	
	91574	0	
	90839	3	
	58235	5	
% Efficacy		94.9	
*P*-value		< 0.0001	

### Faecal *Angiostrongylus vasorum* first-stage larval counts

While no larvae were recovered from the faeces collected on Day 35 (i.e. 42 days after first inoculation), two dogs from the untreated group excreted one and three larvae per 10 g of faeces collected on Day 49 (i.e. 56 days after first inoculation). Overall, *A. vasorum* L1 larvae were recovered from the faeces collected on Days 49, 63, 77 and 90, of 2/10, 6/10, 9/10 and 10/10 untreated dogs respectively. Larval counts of the untreated dogs increased over the course of the study and ranged between 2 and 2819 larvae per 10 g of faeces collected on Day 90 (geometric mean, 133.5 larvae/10 g). In contrast, *A. vasorum* L1 larvae were only recovered from faeces collected on Days 63 and 90, of 1/10 and 2/10 NexGard Spectra®-treated dogs with counts ranging between 1 and 25 larvae per 10 g of faeces collected on Day 90 (geometric mean, 0.5 larvae/10 g).

**Fig. 1:**
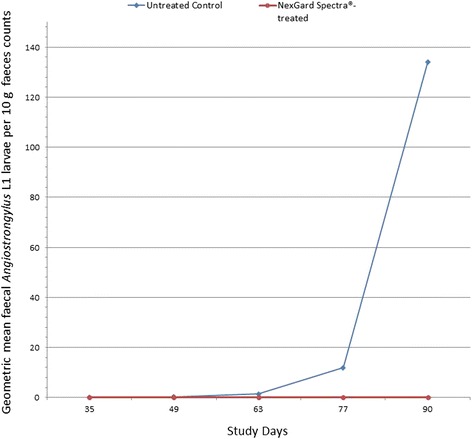
Geometric mean of faecal *Angiostrongylus vasorum* L1 larval counts (study days 35 to 90)

Geometric mean faecal larval counts over the course of the study are shown in Fig. 1. Total cumulative larval output, which is represented by the area under the curves, was reduced by 99.9 % in the NexGard Spectra®-treated dogs compared with the untreated animals (1,277.2 larvae/10 g*day *vs* 1.1 larvae/10 g*day; *F*_(1,9)_ = 65.46, *P* < 0.0001).

### Clinical signs and gross pathology lesions

Physical examination with special regard to cardiorespiratory signs by determination of heart and respiration rates, auscultation of the lungs, and inspection of mucous did not reveal any abnormality in any dog and there was no difference in body weight gain between the dogs of the two groups over the course of the study.

*In*-*situ* inspection of the lungs in open thoracic cavity (prior to reverse perfusion for recovery of the worms) revealed that lungs of the dogs from the treated group had a normal external appearance consisting of pink, uniform, smooth surface normal lungs with limited circumscribed lesions whereas lungs of the dogs in the untreated dogs displayed changes of the surface consisting of overall pale beige to brownish yellow areas often associated with multifocal dark red patches and spots, and marbled and hardened surface consistent with multifocal hemorrhages areas and granulomatous pneumonia.

**Fig. 2:**
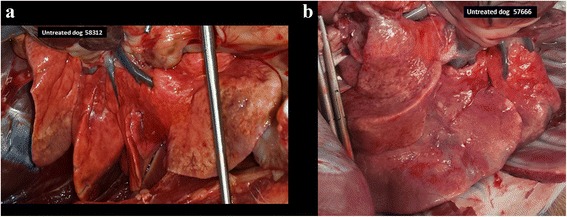
**a**, **b** Lungs from two dogs of the untreated control group

**Fig. 3:**
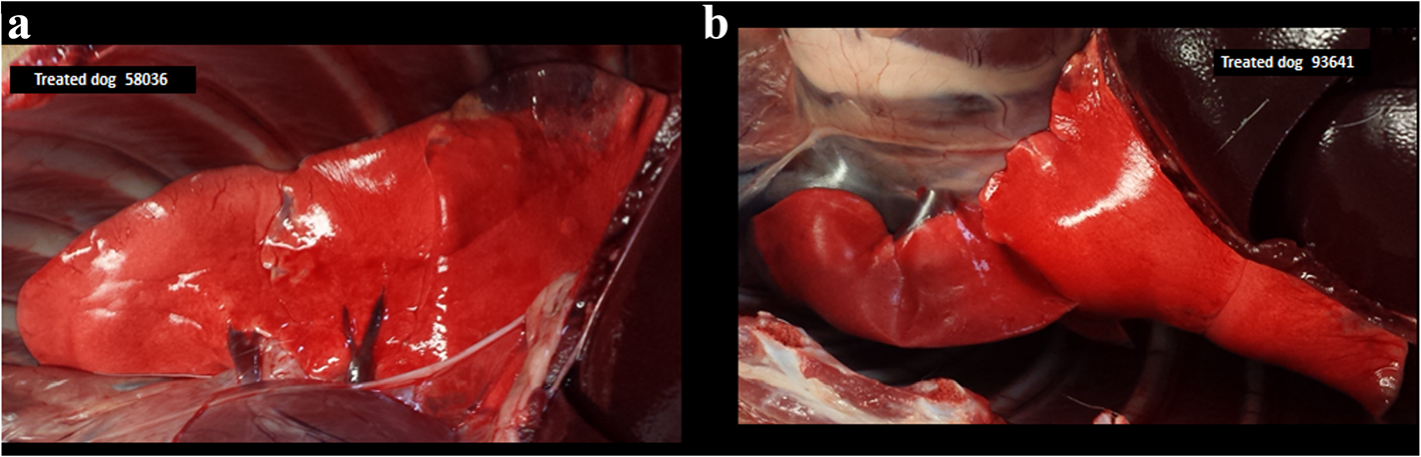
**a**, **b** Lungs from two dogs of the NexGard Spectra®-treated group

Representative images of lungs of untreated and treated dogs are presented in Figs. 2 and 3, respectively.

## Discussion

Milbemycin oxime has been used in the treatment and control of canine nematode infections since the early 1990s. Previously reported controlled laboratory and multi-center field studies testing the efficacy of the oral afoxolaner plus milbemycin oxime chewable tablet formulation NexGard Spectra® administered once to dogs have confirmed that milbemycin oxime provides high efficacy against a broad range of canine adult intestinal nematode infections and prevents heartworm disease [21–24]. Results of this study have demonstrated that monthly administration of NexGard Spectra® to dogs is highly efficacious in the prevention of *A. vasorum* infection. This is in accordance with an epidemiological study conducted in an endemic area in the UK, where anthelmintic treatment, specifically milbemycin oxime, given in the 1–12 weeks before sampling appeared to decrease the chance of testing positive for *A. vasorum* [15].

The multiple challenge infection (trickle infection) model used in this study was designed to better mimic the natural conditions of infection of dogs. Studies assessing the presence of *A. vasorum* in gastropod molluscs have indeed reported not only highly variable prevalence [25] but also a large variation in the burden of *A. vasorum* larvae (range 1–743 per gastropod [12], with most gastropods harbouring low numbers [12, 26, 27]). Thus, under natural conditions dogs are likely to get infection through repeated exposure to small numbers of infectious *A. vasorum* larvae. On average 26.7 % of the total inoculum size was recovered as adult and immature *A. vasorum* from the untreated animals at necropsy in this study. This infection rate as well as the larval excretion eight weeks after the initial inoculation is within the range of findings from recently reported single point challenge studies [12, 14, 28]. The short interval between the latest inoculation and the necropsy was sufficient to allow the molt of larval stages into immature adults. Indeed, Rosen et al. [29] showed that *A. vasorum* complete their 4th molt (L4 to immature adult) within seven days after infection in the abdominal lymph nodes of dogs and that 30 % of *A. vasorum* immature adults were recovered from the right heart/pulmonary arteries within ten days following infection and 80 % within 14 days following infection [29]. In this latest study, dogs were younger than in the present one (1.5 to 3.5 months old) but the difference in the age seems not to have an impact on the development of *A. vasorum* since egg shedding (i.e. development of gravid females) started at a similar time in both studies (Day 49 in Rosen study; between Day 42 and Day 56 in the present study). Since the interval between last inoculation and necropsy was 13–15 days in the present study, the majority of inoculated nematodes have reached the circulatory system where they can be readily recovered. In addition, as efficacy calculation is based on the comparison between numbers of nematodes recovered from untreated controls and treated groups, both of which submitted to similar conditions of infection and necropsy, therefore there is no bias in the results of efficacy.

Prevention of establishment of *A. vasorum* in the treated dogs compared to the untreated animals by almost 95 % was accompanied by a reduction of faecal larval shedding of 99.9 %. Thus, monthly administration of NexGard Spectra® to dogs in *A. vasorum* endemic areas has the potential to reduce in magnitude the contamination of the environment with infectious stages for intermediate hosts which may contribute to lower the risk of disease transmission.

According to the Medical Agencies worldwide (Europe, USA, Japan, Australia), the requirements for approval of an anthelmintic product are based on significant statistical differences between the treated and control groups and on calculated percent effectiveness of 90 % or more (EMEA-CVMP/VICH/832/99, 2000). Previous studies evaluating the efficacy of milbemycin oxime (0.75–1.18 mg/kg body weight) [14], or moxidectin (2.5 mg/kg) [12] against the establishment of angiostrongylosis have demonstrated respectively 98.8 and 100 % reduction of *A. vasorum* counts in the lung of treated animals. In those studies, a single treatment was performed respectively two days or 30+/−2 days post a single inoculation of 200–250 larvae [12, 14]. These results, along with those of the present study, corroborate the hypothesis that monthly administration of lactone macrocyclic would reduce the risk of canine angiostrongylosis.

No clinical sign indicative for angiostrongylosis was observed during clinical examination of any of the animals from both groups. A similar asymptomatic pattern in experimentally infected animals has been previously described in another anthelmintic study [12] where *A. vasorum* counts in untreated dogs were comparable to the numbers recovered from the untreated dogs in the present study. In two other experimental studies, dogs presented respiratory signs [12, 30] but those signs were only noticed in very few control animals in one of those studies [12]. The expression of clinical signs seems therefore to be variable in experimental infection with *A. vasorum*. The absence of clinical signs in the untreated dogs in this study may be related to the short observation period of 14 weeks following initial inoculation but may also be related to the limited exercise of the dogs kept in laboratory conditions.

## Conclusion

In conclusion, the results of this study demonstrate that monthly administrations of NexGard Spectra® is an effective treatment for the prevention of canine *A. vasorum* infection in addition to the proved efficacy of the chewable tablet formulation in the treatment and control of infections with major intestinal nematodes, fleas and ticks [18, 31] and prevention of heartworm disease [18]. Administration of NexGard Spectra® offers a convenient solution for the treatment of dogs at risk of multi-parasitic infections. As a palatable oral product, NexGard Spectra® is well suited to favour the medication adherence which is a critical factor in preventive control programs [32].

## References

[1] Morgan ER, Shaw SE, Brennan SF, De Waal TD, Jones BR, Mulcahy G (2005). *Angiostrongylus vasorum*: a real heartbreaker. Trends Parasitol.

[2] Schnyder M, Schaper R, Bilbrough G, Morgan ER, Deplazes P (2013). Seroepidemiological survey for canine angiostrongylosis in dogs from Germany and the UK using combined detection of *Angiostrongylus vasorum* antigen and specific antibodies. Parasitology.

[3] Elsheikha HM, Holmes SA, Wright I, Morgan ER, Lacher DW (2014). Recent advances in the epidemiology, clinical and diagnostic features, and control of canine cardio-pulmonary angiostrongylosis. Vet Res.

[4] Van Doorn DC, van de Sande AH, Nijsse ER, Eysker M, Ploeger HW (2009). Autochthonous *Angiostrongylus vasorum* infection in dogs in The Netherlands. Vet Parasitol.

[5] Alho AM, Schnyder M, Schaper R, Meireles J, Belo S, Deplazes P, de Carvalho LM. Seroprevalence of circulating *Angiostrongylus vasorum* antigen and parasite-specific antibodies in dogs from Portugal. (In press in Parasitol Res). 10.1007/s00436-016-5001-x PMC4914520 27000086

[6] Kirk L, Limon G, Guitian FJ, Hermosilla C, Fox MT (2014). *Angiostrongylus vasorum* in Great Britain: a nationwide postal questionnaire survey of veterinary practices. Vet Rec.

[7] Serres E (1854). Entozoaires trouvés dans l’oreille droite, le ventricule correspondant et l’artère pulmonaire d’un chien. J Vet Midi.

[8] Bolt G, Monrad J, Frandsen F, Henriksen P, Dietz HH (1993). The common frog (*Rana temporaria*) as a potential paratenic and intermediate host for *Angiostrongylus vasorum*. Parasitol Res.

[9] South A (1992). Terrestrial slugs: biology, ecology, and control.

[10] Anderson RC (2000). Nematode parasites of vertebrates. Their development and transmission.

[11] Koch J, Willesen JL (2009). Canine pulmonary angiostrongylosis: An update. Vet J.

[12] Schnyder M, Fahrion A, Ossent P, Kohler L, Webster P, Heine J, Deplazes P (2009). Larvicidal effect of imidacloprid/moxidectin spot-on solution in dogs experimentally inoculated with *Angiostrongylus vasorum*. Vet Parasitol.

[13] Schnyder M, Fahrion A, Riond B, Ossent P, Webster P, Kranjc A, Glaus T, Deplazes P (2010). Clinical, laboratory and pathological findings in dogs experimentally infected with *Angiostrongylus vasorum*. Parasitol Res.

[14] Böhm C, Schnyder M, Thamsborg SM, Thompson CM, Trout C, Wolken S, Schnitzler B (2014). Assessment of the combination of spinosad and milbemycin oxime in preventing the development of canine *Angiostrongylus vasorum* infections. Vet Parasitol.

[15] Morgan ER, Jefferies R, van Otterdijk L, McEniry RB, Allen F, Bakewell M, Shaw SE (2010). *Angiostrongylus vasorum* infection in dogs: presentation and risk factors. Vet Parasitol.

[16] Helm JR, Morgan ER, Jackson MW, Wotton P, Bell R (2010). Canine angiostrongylosis: an emerging disease in Europe. J Vet Emerg Crit Care.

[17] Vercruysse J, Holdsworth P, Letonja T, Barth D, Conder G, Hamamoto K, Okano K (2001). International harmonisation of anthelmintic efficacy guidelines. Vet Parasitol.

[18] Vercruysse J, Holdsworth P, Letonja T, Conder G, Hamamoto K, Okano K, Rehbein S (2002). International harmonisation of anthelmintic efficacy guidelines (Part 2). Vet Parasitol.

[19] Jacobs DE, Arakawa A, Courtney CH, Gemmell MA, McCall JW, Myers GH, Vanparijs O (1994). World Association for the Advancement of Veterinary Parasitology (W.A.A.V.P.) guidelines for evaluating the efficacy of anthelmintics for dogs and cats. Vet Parasitol.

[20] Ministry of Agriculture, Fisheries and Food (1986). Manual of Veterinary Parasitological Laboratory Techniques.

[21] EMA. European Medicines Agency. NexGard Spectra Product information EPAR. http://www.ema.europa.eu/docs/en_GB/document_library/EPAR_-_Summary_for_the_public/veterinary/003842/WC500181964.pdf (2015). Accessed 03 March 2016

[22] Fankhauser B, Hamel D, Dorr P, Reinemeyer CR, Crafford D, Bowman DD, et al. Efficacy of oral afoxolaner plus milbemycin oxime chewables against induced infections of gastrointestinal nematodes in dogs. Vet Parasitol. 2016; in press. 10.1016/j.vetpar.2016.06.003 27369586

[23] Rehbein S, Dorr P, Bowman DD, Crafford D, Kusi I, Postoli R (2016). Efficacy of oral afoxolaner plus milbemycin oxime chewable tablets against naturally acquired infections of gastrointestinal nematodes in dogs. Vet Parasitol.

[24] Rehbein S, Knaus M, Mallouk Y, Breiltgens T, Brianti E, Capári B, et al. Efficacy against nematode infections and safety of afoxolaner plus milbemycin oxime chewable tablets in domestic dogs under field conditions in Europe. Vet Parasitol. (in press). 10.1007/s00436-016-5287-8 27771803

[25] Aziz NAA, Daly E, Allen S, Rowson B, Greig C, Forman D, Morgan ER (2016). Distribution of *Angiostrongylus vasorum* and its gastropod intermediate hosts along the rural–urban gradient in two cities in the United Kingdom, using real time PCR. Parasit Vectors.

[26] Ferdushy T, Kapel CM, Webster P, Al-Sabi MN, Grønvold J (2009). The occurrence of *Angiostrongylus vasorum* in terrestrial slugs from forests and parks in the Copenhagen area, Denmark. J Helminthol.

[27] Patel Z, Gill AC, Fox MT, Hermosilla C, Bckeljau T, Breugelmans K, Keevash E, McEwan C, Aghazadeh M, Elson-Riggins JG (2014). Molecular identification of novel intermediate host species of *Angiostrongylus vasorum* in Greater London. Parasitol Res.

[28] Willesen JL, Kristensen AT, Jensen AL, Heine J, Koch J (2007). Efficacy and safety of imidacloprid/moxidectin spot-on solution and fenbendazole in the treatment of dogs naturally infected with *Angiostrongylus vasorum* (Baillet, 1866). Vet Parasitol.

[29] Rosen L, Ash LR, Wallace GD (1970). Life history of the canine lungworm *Angiostrongylus vasorum* (Baillet). Am J Vet Res.

[30] Kranjc A, Schnyder M, Dennler M, Fahrion A, Makara M, Ossent P (2010). Pulmonary artery thrombosis in experimental *Angiostrongylus vasorum* infection does not result in pulmonary hypertension and echocardiographic right ventricular changes. J Vet Intern Med.

[31] Rehbein S, Fourie JJ, de Vos C, Anderson A, Larsen DL, Jeannin P (2016). Efficacy of oral afoxolaner plus milbemycin oxime chewables against induced infestations with *Dermacentor reticulatus* in dogs. Parasitol Res.

[32] World Health Organization (2003). Adherence to long-term therapies: evidence for action.

